# The Role of a Mentorship Program on the Relationship between Neglect and Depression among Adolescents in Low-Income Families

**DOI:** 10.3390/ijerph18137010

**Published:** 2021-06-30

**Authors:** Jaewon Lee, Jennifer Allen, Hyejung Lim, Gyuhyun Choi, Jiyu Jung

**Affiliations:** 1 Department of Social Welfare, Inha University, Incheon 22212, Korea; j343@inha.ac.kr; 2 School of Social Work, Michigan State University, East Lansing, MI 48823, USA; allenj66@msu.edu; 3 School of Education, Korea University, Seoul 02841, Korea; nanapro@korea.ac.kr; 4 Integrative Arts Therapy, Dongduk Women’s University, Seoul 02748, Korea; toyou4048@uos.ac.kr; 5 Korea Development Bank Foundation, Seoul 07242, Korea

**Keywords:** mentorship program, low-income students, neglect, depression

## Abstract

This study examines the moderating effect of a mentorship program on the relationship between parental neglect and depression among adolescents from low-income households since COVID-19. A total of 264 participants from all provinces in South Korea were registered for a mentorship program provided by the Korea Development Bank [KDB] Foundation, which is a charitable and non-profit organization. Two-hundred fifty-five middle and high school students from low-income families were included in the final sample. The mentorship program was provided to students based on mentors’ advice and feedback. A bootstrap method using the PROCESS macro 3.4 for SPSS was utilized to examine the moderating effect of satisfaction with the mentorship program. Neglect was positively related to depression among low-income students. Satisfaction with the mentorship program moderated the relationship between low-income students’ neglect and depression. Visits from social workers or other advocates or volunteers to low-income families with children may be helpful to address depression among low-income students. High quality mentorship programs should be provided to more low-income students for their mental health, funded particularly in the context of corporate social responsibility. Particularly since the COVID-19 pandemic, financial contributions by corporations would be valuable to reconstruct the damage to quality of life and psychological well-being among low-income adolescents.

## 1. Introduction

Depression is a common mental health issue experienced by adolescents in South Korea (e.g., [1,2,3]). Researchers have found that one contributing factor for high levels of adolescent depression is neglect by parents or caregivers [4,5,6,7]. One intervention that has been found to be successful in reducing levels of depression among high-risk adolescents are formal mentorship programs, or programs where one person (the mentee) is matched with another person (the mentor) who provides support in various ways (e.g., [8,9]). Formal mentorship programs are distinguished from informal mentorship programs by the fact that formal programs have a set of guidelines or a process in which the mentorship takes place, while informal mentoring is more unstructured [10]. Some aspects of effective formal mentorship programs include consistent meetings between mentor and mentee and open communication between mentor and mentee [10,11]. Such mentorship programs may be particularly beneficial for adolescents with neglectful parents and caregivers, as such programs provide them with an adult who takes a supportive and structured role in their lives. However, more research is needed on the moderating role of participation in a mentorship program on the relationship between adolescents’ experiences of parental neglect and adolescents’ depression.

## 2. Literature Review

### 2.1. Depression among Low-Income Adolescents

A few studies have examined the relationship between income or socioeconomic status and depression among adolescents in South Korea and findings have been mixed as to the association between depression and household income or socioeconomic status [2,12,13]. Data from a 2006 survey of Korean youth indicated that adolescents with a lower socioeconomic status were more likely than adolescents with a high socioeconomic status to experience depression [2]. However, data from another national survey of Korean youth revealed no significant differences in adolescent depression depending on their household’s monthly income [12]. Additionally, in one sample of more than 75,000 middle- and high school students, 43.96% of girls and 32.03% of boys reported experiencing depressive symptoms in the past year, and students in high-income households reported worse depression than students in lower income households [13]. Despite these mixed findings on the association between socioeconomic status and depression, prevalence of depression has been found to be fairly common among South Korean adolescents, with prevalence rates ranging from across a number of studies [1,3,14]. Of note, the recent COVID-19 pandemic has also contributed to increased depression among adults in South Korea and adolescents in other countries, including Australia, China, and the United States [6,15,16,17]. Such increases in depression have been particularly evident among low-income adolescents or among adolescents whose parents have experienced a job loss [18,19,20]. Increases in depression during the COVID-19 pandemic may be associated with reduced contact with friends and extended family, a reduction in physical activity or participation in sports, difficulties with virtual schooling, boredom, and fear of coronavirus infection, for example [15,16,18,19].

### 2.2. Negative Effects of Parental Neglect during Adolescence

Researchers have found that experiencing parental neglect during adolescence is positively associated with adolescents’ depression [4,5,6,7]. First, in a study of multicultural adolescents living in South Korea with a Korean father and a non-Korean mother, adolescents who reported that their parents engaged in a neglectful parenting style had higher depression than those who did not [6]. Further, in a longitudinal assessment of the effect of childhood and adolescent neglect and abuse on adolescent and adult depression, the researchers found that American adolescents who had experienced neglect were 2.49 times more likely to be diagnosed with major depression than those who did not experience neglect [4]. Additionally, among a large sample of U.S. adolescents with a current case with Child Protective Services, researchers found a significant impact of parent or caregiver neglect on adolescents’ depression [5]. Last, in a rapid systematic review of the research on adolescent neglect published between 1990 and 2014, nine of the thirteen included studies found that neglect was positively associated with internalizing symptoms, including depression [7].

### 2.3. Positive Effects of Mentorship Programs during Adolescence

Mentorship programs have been found to have various benefits for adolescent mentees (e.g., [9,21,22]). In an evaluation of a community-based intervention that included mentorship and weekly meetings, among the 10–12-year-old girls who attended, they showed improvements in self-esteem, peer bonding and school attachment [23]. A systematic review of mentorship programs targeted at young people with disabilities revealed that such programs had positive outcomes related to academic achievement and employment outcomes [21]. In another systematic review of mentorship programs in New Zealand, researchers found that 86% of such programs were effective at reaching their goals relating to psychological outcomes, and 73% met their goals relating to youth’s interpersonal outcomes [24].

Additionally, mentorship programs specifically have been found to affect participating adolescents’ depression [8,9,22]. In a school-based mentorship program that paired U.S. elementary and middle school students with learning disabilities and/or attention-deficit hyperactivity disorder [ADHD] with mentors (high school and college students who also had learning disabilities and/or ADHD) showed that participating students had decreased depression after participation in the program compared to baseline, while students in the control group experienced increases in depression over the period of the mentorship program [8]. Moreover, in a study of Swedish eighth graders, students who participated in structured after-school activities (defined as activities led by a competent adult, with regular meetings and a skill building emphasis) had significantly lower depression than adolescents who did not participate in such an activity, and this was particularly true if the adolescent reported a high level of support from the adult running the activity [22]. Further, participation in an after-school activity was associated with lower depression among adolescents who reported a detached relationship with their parents, characterized by feeling distant or not spending much time together [8,22]. Last, 12–15-year-old at-risk U.S. youths who participated in a program that assigned the youth to a trained adult mentor in the community showed a significant decrease from pre–post levels of depression [9].

### 2.4. The Current Study

Researchers have found that depression is common in adolescence, particularly among those from low-income households [2,14]; that parental neglect in adolescence is associated with increased depression (e.g., [6]); and that participation in a mentorship program is associated with decreased depression (e.g., [9,22]). Despite this, little research has examined the moderating role of participation in a mentorship program on the relationship between parental neglect in adolescence and adolescents’ depression, particularly in the South Korean context, nor has there been a great emphasis on the effect of adolescents’ satisfaction with the mentorship program on these relationships. At the time this study was conducted, in April 2021, the world was still combatting the COVID-19 pandemic; however, in South Korea, schools were partially reopened beginning in June 2020, did not have a full-scale lockdown, and had a comparatively low mortality and case rate compared with other countries [25,26,27]. With this in mind, in this study we address the following research question: Among adolescents from low-income households participating in a mentorship program, does satisfaction with the mentorship program moderate the relationship between parental neglect and depression?

## 3. Methods

### 3.1. Participants and Sampling

Participants were middle and high school students in South Korea. Participating students were limited to those who had grown up in low-income households. The Korea Development Bank [KDB] Foundation, which is a charitable and non-profit organization, recruited middle and high school students in low-income families to provide a mentorship program. Yearly poverty income guidelines were employed to determine if they were low-income students. A total of 264 participants from all provinces in South Korea were registered for the KDB Foundation’s mentorship program. Data collection occurred in April 2021, and the questionnaire was provided via Google Forms. We sent a text message to participants with a link to the online survey. Experts in working with low-income students, such as a social worker in the KDB Foundation and a middle school teacher, reviewed the questionnaires to refine the questions and to protect students’ rights. Students who engaged in the online survey were given a $5 gift card as a reward. On average, it took about twenty minutes for students to finish the survey. A consent form was provided before the online survey and required consent from both students and their caregivers. Some students did not participate in the survey because either they refused to consent, or their caregivers did. Further, other students who we could not reach due to wrong contact information were excluded. Thus, a total of 255 respondents were included in the final sample. As this survey did not collect any private information such as name, address, etc., and did not have any risks to participants, the Institutional Review Board approved the current study (#210216-2A).

### 3.2. Measures

*Depression.* Respondents were asked to report levels of depression. This study used a short form of the Center for Epidemiologic Studies Depression Scale [CES-D] to measure participants’ depression. The measurement consists of seven items: “I did not feel like eating; my appetite was poor;” “I had trouble keeping my mind on what I was doing;” “I felt depressed;” “I felt that everything I did was an effort;” “My sleep was restless;” “I felt sad;” and “I could not get going.” A four-point Likert-type scale was employed, and response options ranged from “rarely or none of the time” to “most or all of the time.” The score of all items was summed and higher scores indicate higher levels of depression. Cronbach’s α of the CES-D scale in this study was 0.81 (*M* = 5.71; *SD* = 4.32; ranging from 0 to 21).

*Neglect.* Neglect in this study refers to how much middle and high school students have experienced neglect by their parents or caregivers. The Parent–Child Conflict Tactics Scale [CTSPC] developed by Straus, Hamby, Finkelhor, Moore, and Runyan [28] was utilized to measure neglect of students by parents or caregivers. The five-item neglect scale, one of subscales of the CTSPC, was provided to students. In the original scale, parents or caregivers were asked to report neglect, but in this study, we had the child or adolescent report neglect. The five items of this subscale include: “I had to be home alone, even when some adult should be with me;” “My parents or caregivers were so caught up with their problems that they were not able to show or tell me that they loved me”, “My parents or caregivers were not able to make sure I got the food I needed;” “My parents or caregivers were not able to make sure I got to a doctor or hospital when I needed it;” and “My parents or caregivers were so drunk or high that they had a problem taking care of me.” Response options in this study were: 1 = This has never happened; 2 = Not in the past year, but it happened before; 3 = 1–2 times in the past year; 4 = 3–5 times in the past year; 5 = Once a month; 6 = 2–3 times a month; and 7 = More than once a week. Each item was summed, and higher scores mean higher levels of neglect. The neglect scale had a Cronbach’s alpha of 0.82 (*M* = 7.69; *SD* = 4.72; ranging from 5 to 35).

*Satisfaction with the mentorship program.* Respondents were asked to answer the extent to which they were satisfied with the quality and benefits of the mentorship program. The mentorship program provided by the KDB Foundation provides one-on-one mentoring by matching mentees with a mentor. Low-income students selected by the KDB foundation have the mentorship program until they graduate high school if they would not refuse to the mentorship program. Generally, mentees were recruited at the beginning of academic year. Mentors include social workers, teachers, professionals who specialize in psychology, etc., and mentees were middle or high school students in low-income families.

Based on a guide provided the KDB Foundation for the mentoring program, the mentors encourage students to develop their abilities for future careers and increase psychological well-being through mutual communication. In-person communication with a mentor was available at least once a month, but online formats, including phone call or webinars, were an alternative way for the meeting due to COVID-19. Generally, the meeting lasts more than two hours; however, it is flexible depending on students’ needs. Thus, the mentorship program provides in-person meetings at least once a month, as well as continuous interactions between mentees and mentors to help middle and high school students’ psychological health and to support their developmental achievement. In other words, mentors’ advice and feedback based on mutual communication play an important role to buffer against anti-social behaviors and juvenile delinquency.

Additionally, beyond the general mentorship program that every student received, extra mentoring was provided that was tailored to the students’ age, for example. The extra mentoring program consists of three different contents based upon age and grade: a program improving quality of relationships for middle school students; a program improving career maturity for high school students in the first grade; and a program experiencing artistic and cultural activities for high school students in second and third grade. To measure how satisfied low-income students were with the mentorship program, they responded to seven items: “I am satisfied with the relationship with my mentor”; “My mentor helps me to achieve my developmental tasks”; “The mentorship program improves the quality of my life”; “The mentorship program is beneficial for my psychological health”; “I am satisfied with the frequency of in-person or online meetings with my mentor”; “I am satisfied with the frequency of the extra mentoring tailored for me”; and “I am satisfied with the quality of the extra mentoring tailored for me”. Each item was rated on a five-point Likert-type scale with response options ranging from 1 (strongly disagree) to 5 (strongly agree). All items were summed, and higher scores referred to greater satisfaction with the mentorship program. The Cronbach’s α of the five-point Likert-type scale was 0.94 (*M* = 31.03; *SD* = 4.59; ranging from 15 to 35).

### 3.3. Control Variables

Respondents’ gender, age, and academic performance were assessed, and their parents’ higher education attainment was also considered. If both mothers and fathers received higher education, they were classified into higher education. 

### 3.4. Analysis Strategies

Descriptive analysis was used to describe the descriptive statistics used in this study. The PROCESS macro 3.4 for Statistical Product and Service Solutions (SPSS) was employed to identify the hypothesis that satisfaction with the mentorship program moderated the relationship between neglect and depression among middle and high school students from low-income families. A bootstrap method was utilized to examine the moderating effect of satisfaction with the mentorship program. Five-thousand iterations of the bootstrapping method were performed at the 95% bootstrap confidence intervals. 

## 4. Results

Descriptive statistics are shown in Table 1. The average scores of depression and neglect among low-income students were 5.71 and 7.69, respectively. Average satisfaction with the mentorship program was 31.03. About half of total respondents were girls (49.4%) and respondents’ average age was 17.36. Given cultural differences in South Korea, their international age was 16.36 years old, as a newborn starts at one year old in Asian countries. The average score for academic performance was 7.66, indicating that the average letter grade in primary classes was about C. For parental educational attainment, 39.6% of respondents’ parents, including both mothers and fathers, received higher education.

**Figure 1:**
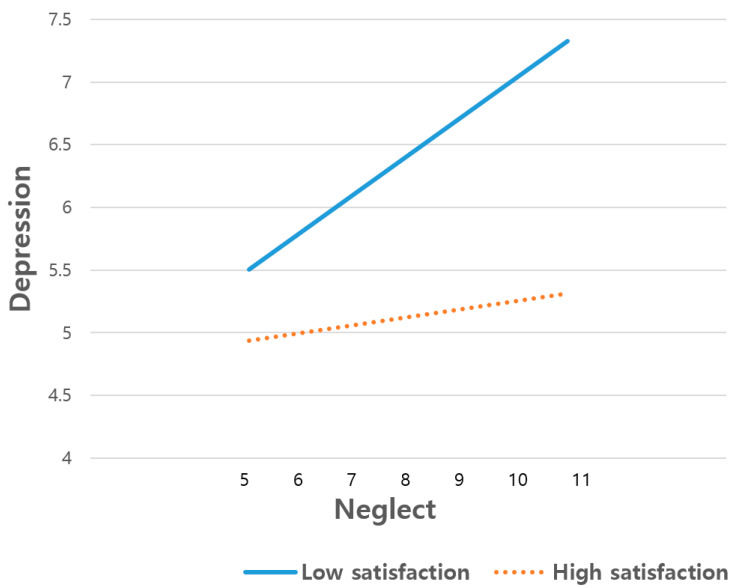
Moderating effect of satisfaction with the mentorship program on the relationship between neglect and depression among low-income students.

Table 2 shows the moderating effect of satisfaction with the mentorship program on the association between neglect and depression among students in low-income families. The moderating effect of satisfaction with the mentorship program was statistically significant (β = −0.03, *p* < 0.05). In other words, satisfaction with the mentorship program moderated the relationship between students’ neglect and depression. Additionally, we found that neglect was positively related to depression, accounting for age, gender, academic performance and parental education (β = 1.00, *p* < 0.01) and parents’ higher education was negatively associated with depression (β = −1.44, *p* < 0.01). Figure 1 indicated the moderating effect of satisfaction with the mentorship program on the relationship. Regardless of how satisfied middle and high school students in low-income families were with the mentorship program, those who experienced low levels of neglect also reported low levels of depression. However, students who were more satisfied with the mentorship program showed lower levels of depression compared to those who were less satisfied with the mentorship program, regardless of how much they had experienced neglect by their parents (4.94 for the more satisfied group vs. 5.51 for the less satisfied group; 5.32 for the more satisfied group vs. 7.33 for the less satisfied group). That is, depression among low-income students was greatly influenced by levels of satisfaction with the mentorship program. In particular, if students frequently encounter neglect by their parents, the effect of satisfaction with the mentorship program was greater, showing a 1.82 score gap in depression among the less satisfied group, while there was a 0.38 score gap among the more satisfied group.

## 5. Discussion and Conclusions

This study explored the relationship between neglect and depression among low-income students since COVID-19, as well as the moderating effect of satisfaction with a mentorship program on the relationship. We found that low-income middle and high school students who have been exposed to more neglect by their parents were also more likely to be depressed. Further, satisfaction with the mentorship program moderated the relationship between neglect and depression among students from low-income families. Therefore, during the COVID-19 pandemic, additional programs such as a mentorship program operated by a non-profit organization play an important role to relieve mental health problems among students from low-income families.

This study revealed that neglect is positively related to depression, which is consistent with previous research [4,5,6,7]. Given that the period from middle school to high school is an important period for psychological development ([29]), middle school and high school students who experience more neglect by their parents are more likely to be at risk of depression. Thus, this study sheds light on why neglect by parents is an important issue related to mental health among adolescents in low-income families since COVID-19. For students in low-income families, their parents may not have enough time to take care of their children, because they have to work many hours to support the family [30]. Considering this study investigates this relationship since COVID-19, both mothers and fathers in low-income households may be forced to spend more time in the workplace than before COVID-19 due to the related economic recession [31]. As a result, children in low-income families are more likely to experience neglect since COVID-19, during a time when they need more care from parents as they are spending increased time at home because of hybrid or online classes. As such, adolescents need more protection and care since COVID-19; however, their parents might have reduced time to care for their children, leading to more neglect. Thus, middle and high school students who were exposed to more neglect reported more depression [4,5,6,7]. In addition, the COVID-19 pandemic has greatly affected humans worldwide, including increased stress [32,33]. Parents who experience more stressors in their workplaces and in daily life because of the negative effects of COVID-19 might pay less attention to their children as they are too exhausted. Therefore, it is particularly critical to pay attention to adolescents in low-income families, as they might be particularly affected by relatively limited resources and low family support than higher income households [34]. During the COVID-19 crisis, parents in low-income families may be forced to work more in order to maintain the same financial quality of life as before COVID-19. Thus, increased neglect occurring in low-income families during COVID-19 should be addressed by governmental and/or community’ support. Visits from social workers or other advocates or volunteers to low-income families with children may be helpful to address depression among low-income students because they have some time to communicate with others. If in-person visits are not available because of the spread of COVID-19, online meetings, using tools such as Zoom, can be an alternative way to contact students in low-income families.

The significant moderating effect of satisfaction with the mentorship program indicates that providing a high-quality mentorship program is beneficial to address depression among adolescents who experience neglect by their parents. Middle and high school students still need their parents’ support to successfully complete their developmental tasks. However, adolescents in low-income families tend to experience more neglect because their parents may be busy working to support the family. As a result, middle and high school students in low-income families are more likely to be at greater risk of depression. Given that depression is relatively common in adolescence [1,2,14], a mentorship program for low-income students can play an important role against adolescents’ depression because mentors can support their mentees’ mental health. As this study confirmed the moderating effect of satisfaction with the mentorship program, mutual interactions between mentees and mentors may increase levels of satisfaction with the mentorship program, leading to reduced levels of depression among low-income students who experience either more neglect or less neglect.

Specifically, low-income students who were more satisfied with the mentorship program showed lower levels of depression, even if they were exposed to higher levels of neglect. This sheds light on the importance of perceived quality of and satisfaction with the mentorship program. Generally, mentorship programs have been regarded as a good way to support students [9,21,24]. Along with this, this study contributes to understanding how the quality of the mentorship program determines how satisfied participants are with the program, particularly for low-income students. Given that low-income students are more frequently exposed to neglect by parents [35], the role of a mentorship program is important to address mental health among low-income students experiencing neglect by their parents. Thus, high quality mentorship programs should be more provided to low-income students for their mental health, funded particularly in the context of corporate social responsibility [36]. Generally, it is encouraged for government or community centers to offer mentorship programs, but more attention should be given to the role of corporate foundations to better societies and communities and to expand financial support for programs like the mentorship program for low-income students. In particular, during the COVID-19 pandemic, which has greatly deteriorated quality of life among low-income families (e.g., [37]), financial contribution by corporations would be valuable to reconstruct the damaged quality of life and psychological well-being among low-income adolescents.

## 6. Limitations

Although this study contributes to the understanding of the importance of a mentorship program on the relationship between neglect and depression among low-income students, we encourage readers to consider several limitations of the current study. First, perceived quality of the mentorship program may be different in other countries, as these findings are limited to a South Korean context. Further, characteristics of low-income students and other cultural differences should be considered when interpreting these findings to apply to other cultures. Second, using a self-reported survey may bring about a social desirability bias. Thus, responses particularly regarding neglect by parents may be higher in actuality than our results indicate.

## Figures and Tables

**Table 1:** Descriptive Statistics.

Variables	% or Mean (SD)
Depression	5.71 (4.32)
Neglect	7.69 (4.72)
Satisfaction with the mentorship program	31.03 (4.59)
Gender (girl)	49.4%
Age	17.36 (1.75)
Academic performance	7.66 (3.73)
Parent’s higher education	39.6%

**Table 2:** Regression results of unstandardized coefficients (standard error) predicting depression.

Variables	Depression	
(Constant)	1.95 (4.08)	
Neglect	1.00 (0.35)	**
Gender (girl)	0.68 (0.52)	
Age	0.06 (0.15)	
Academic performance	−0.09 (0.07)	
Parent’s higher education	−1.44 (0.54)	**
**Moderator**		
Satisfaction with the mentorship program	0.07 (0.11)	
**Moderating effect**		
Neglect * Satisfaction with the mentorship program	−0.03 (0.01)	*

Note. * *p* < 0.05. ** *p* < 0.01.

## Data Availability

The data presented in this study are available on request from the corresponding author. The data are not publicly available due to ethical reasons.
